# Genetic risk score to improve prediction and treatment in gestational diabetes mellitus

**DOI:** 10.3389/fendo.2022.955821

**Published:** 2022-10-19

**Authors:** Yumeng Tian, Ping Li

**Affiliations:** Department of Endocrinology, Shengjing Hospital of China Medical University, China Medical University, Shenyang, China

**Keywords:** diabetes mellitus, type 2 diabetes mellitus, single-nucleotide polymorphism, genetic risk score, endocrinology

## Abstract

Diabetes mellitus is a chronic disease caused by the interaction of genetics and the environment that can lead to chronic damage to many organ systems. Genome-wide association studies have identified accumulating single-nucleotide polymorphisms related to type 2 diabetes mellitus and gestational diabetes mellitus. Genetic risk score (GRS) has been utilized to evaluate the incidence risk to improve prediction and optimize treatments. This article reviews the research progress in the use of the GRS in diabetes mellitus in recent years and discusses future prospects.

Gestational diabetes mellitus (GDM) refers to varying degrees of glucose intolerance that first appear during pregnancy (1). It has been associated with poor maternal and offspring outcomes, including preeclampsia, polyhydramnios, operative delivery, shoulder dystocia, birth canal lacerations, and fetal overgrowth (also called macrosomia) (2). The patient’s blood glucose is normal before pregnancy, but during pregnancy, blood glucose quietly and gradually increases, which may cause adverse effects on the mother and the baby before diagnosis and recognition. Therefore, early prediction of GDM has been explored to facilitate the prevention, early diagnosis, and treatment of the disease. Currently, the pathogenesis of GDM is believed to be closely related to the glycemic effect of placental hormones, but some studies have shown that GDM has an obvious genetic background and a similar genetic pathway with type 2 diabetes mellitus (T2DM) (3, 4). Therefore, the genetic association between T2DM and GDM has been analyzed in recent years to seek breakthroughs. Genome-wide association studies (GWAS) are one way to do that. GWAS have analyzed at least hundreds of single-nucleotide polymorphisms (SNPs) to determine their associations with complex clinical conditions and phenotypes. Similar to type 2 diabetes, GDM is closely related to both genetic and environmental factors, each of which may increase the risk of developing the disease (5). The effect of a single or small number of SNPs is too weak to accurately predict the disease; thus, the cumulative effect of more SNPs needs to be determined. The genetic risk score (GRS) is not only a common strategy but also a new stage in the detection of genetic susceptibility to complex diseases. The GRS is a useful gene analysis strategy that integrates genetic information from multiple loci to predict an individual’s likelihood of developing a disease (or a specific clinical trait) and to assess the effectiveness of associated variations in predicting the disease. This paper reviews the research status and the application value of the GRS in GDM by summarizing the progress in domestic and foreign research. This paper aims to help clinicians understand the role of genetic background in the pathogenesis of GDM and explore the value of the GRS in the diagnosis and treatment strategy of GDM.

## Introduction to GRS

A GRS is used to evaluate the effect of genetic susceptibility factors in risk prediction models. A GRS allows for the assessment of the contributions of numerous factors to disease development and outcomes, including disease susceptibility, progression, and response to treatment (6). At present, the clinical application of such evaluations is the polygenetic risk score (PRS), which can condense a large amount of genomic variation information into scores that can measure individual susceptibility, calculated as a totality of their genome-wide genotypes and (or) weighted by the corresponding genotype effect size estimates based on GWAS statistical data (7). A PRS is an extension of a large number of GRSs that may be related to markers. It is aimed to predict all genetic variations measured by correlated markers (6). Taking T2DM as an example, the purpose of constructing a GRS model and risk score for the T2DM population is divided into two aspects: 1) to estimate the risk of an individual developing T2DM based on existing information, usually genetic, clinical, demographic, or a combination, or to predict risk only for a disease characteristic, such as insulin resistance, simple fasting blood glucose, and HbA1c; and 2) to assess the predictive power of a single SNP for the risk of T2DM and thus establish the contribution of this SNP to the etiology of T2DM. The prediction accuracy of the GRS is most often valued by measuring the area under the curve (AUC) of the receiving operator characteristic, an indicator of model accuracy (6).

The methods that most studies used included simple count GRS [SC-GRS, sGRS, or unweighted GRS (uGRS)], weighted GRS (wGRS), pruning and thresholding (P + T), LDpred GRS, GraBLD GRS, and direct logistic regression GRS (DL-GRS). The SC-GRS and wGRS are the most commonly used. The wGRS is calculated based on the number of risk alleles weighed by their effect sizes reported in previous GWAS. The SC-GRS is calculated as the sum of the risk alleles (0, 1, 2) of each of the variants. The formulas are shown in **Table 1**.

**Table 1:** The formulas of the three most commonly used GRS.

Category	Calculation	Description
SGRS	$\text{GRS}=\sum_{\text{i}=1}^{\text{I}}{\text{G}}_{\text{i}}$	Calculated as the sum of the risk alleles (0, 1, 2) of each of the variants.
wGRS	${\text{β}}_{\text{i}}=\ln({\text{OR}}_{\text{i}})$ $\text{GRS}=\sum_{\text{i}=1}^{\text{I}}{\text{β}}_{{\text{OR}}_{\text{i}}}{\text{G}}_{\text{i}}$	Calculated based on the number of risk alleles weighed by their effect sizes reported in previous GWAS
DL-GRS	$\text{GRS}=\sum_{\text{i}=1}^{\text{I}}{\text{β}}_{\text{i}}{\text{G}}_{\text{i}}$	The weights were derived from the existing original data, and the logistic regression model was fitted with these data. The SNP effect estimated in the model was used as the weight to calculate the OR weights and formulas of all SNPs included in the model

## GRS used in studies on T2DM

### GRS for predicting the risk for T2DM

GWAS have increasingly identified more SNPs that are related to the risk factors of T2D. Considerable research has demonstrated that the GRS improves the accuracy of T2D risk prediction, including research conducted in Europe, Asia, and North America. A study showed that GRST2D, fasting plasma glucose GRS (GRSFPG), insulin secretion GRS (GRSIS), and 2-h plasma glucose GRS (GRS2hPG) were associated with increased glucose AUC, increased FPG, decreased insulin secretion, and increased 2hPG, respectively (*p*< 0.0017 for all models) (8). Miranda-Lora et al. found that for non-diabetic Mexican Americans, the beta-cell GRS was related to reduced insulinogenic index, the insulin sensitivity GRS was related to the corresponding effect, the lipodystrophy GRS was related to reduced adiposity, and the body mass index plus lipid GRS was related to increased insulin clearance. All GRSs were strongly associated with T2D (9). Another study showed that the GRS of the associated variants of NF-E2-related factor 2 expression is prone to be a useful indicator of T2D development in the normal human population. In addition, linear regression analyses showed positive associations between the GRS and fasting glucose (*p*-value = 0.028, *β* = 0.62), 2-h glucose (*p*-value = 0.0004, *β* = 1.13), and HbA1c (*p*-value = 0.033, *β* = 0.03) (10). Inaishi et al. revealed that among pediatric-onset T2D cases, more than 95% of the cases had six or more risk alleles, the average GRS was higher compared with the controls, and the joint construction of the model may have a highly suggestive effect on the risk of T2D (11). Furthermore, the erythrocyte phospholipid ALA and unweighted GRS can jointly predict the risk of T2D in a Chinese population (12).

Some studies have combined GRSs with other risk factors to predict T2D. For instance, one study found a significant interaction between family history (FH) and lifestyle factors, and T2D genetic risk combined with the GRS and FH had the strongest interaction with lifestyle scores (13). There is a relationship observed between the beta-cell dysfunction GRS and young onset age with the risk of glycemic progression among people with type 2 diabetes, even though a linear relationship was observed between the GRS and the risk of glycemic progression (14). A multiple GRS (AUC: 0.58; *p* = 1.37 × 10^−17^) presented a greater AUC for T2D prevalence in non-obese than in obese individuals (AUC: 0.53; *p* = 1.4 × 10^−4^). At the same time, the obGRS showed a greater AUC for T2D in obese subjects (15). Likewise, Chikowore et al. found that the GRS, which consisted of variants that had significant associations in their population, was significantly associated with increased T2D risk as indicated by an OR of 1.21 (1.02–1.43). Due to the limitations of the GRS in predicting the risk of T2D, there are few clinical applications in the black population of southern Africa (16).

### GRS for predicting the risk of developing T2DM complications

Moreover, GRSs can be used to predict T2D complications and explore their associations with other diseases. A study used the GRS to assess the T2D susceptibility limit based on 41 of the best-known T2D susceptibility variants. Cumulatively, subjects with higher GRSs had an approximately 40% higher risk of developing severe coronary artery disease (CAD) compared with those with lower scores (17). The APN level and the GRS were two independent risk factors for diabetic retinopathy. The GRS was calculated based on 10 SNPs for each subject, and the cumulative effect of genes was observed (18). Another study found that the higher the mother’s GRS, the higher the offspring’s risk of weight gain (19). These studies indicate that the GRS plays a certain role in the prediction of complications of T2D.

## GRS used in studies on GDM

The genetic background of GDM is similar to that of T2D, so the SNPs related to the two diseases and the constructed GRS will be inevitably overlapped. However, as a special period of the body, the abnormal blood glucose metabolism is associated with not only the mother’s diabetes susceptibility but also the secretion of the various hormones from the placenta, which help to maintain blood glucose. Also, the mother’s changes in weight, blood lipid, and intestinal flora and obstetric complications in pregnancy are all closely related to maternal blood glucose metabolism (2).

Therefore, the pathogenesis of GDM is more complicated, and the SNPs involved and the GRS constructed therefrom are also more diversified, forming a genetic background different from T2D. In this paper, the research progress in this field during recent years will be described.

### Relationship between GRS and the risk of GDM

As shown in **Table 2**, Lamri et al. found that for GDM patients in South Asia, the predictive effect of the PRS can increase by integrating GWAS data, and extracting aggregate statistics from an enormous multi-ethnic genome-wide meta-analysis. Participants with the highest PRSs had an increased risk of GDM compared with the other groups (20). Another study used the SC-GRS and DL-GRS to investigate the association between SNPs and the identified risk factors [age and body mass index (BMI)]. The study showed that the GRS of GDM pregnant women was significantly higher than that of the control group (*p*< 0.001) (21). Moen et al. found that SNPs, which have been proven to be associated with hyperglycemia in Norway’s non-pregnant population, also had the ability to predict the risk of hyperglycemia during pregnancy in the pregnant population by analyzing the known and theoretically related variation of the GRS. Furthermore, SNPs, which have been shown to be associated with glucose metabolism parameters in non-pregnant women, are also related to the same glucose metabolism parameters in pregnant women in the pregnant population, albeit with low statistical power (22).

**Table 2:** Genetic risk score in the studies on GDM.

	Publishing time	Racial groups	Sample size	Amounts of SNPs	Methods	Outcome event	Variants selection for GRS	Conclusion
1	2021	Ethnic Russians	1,142	6	SC-GRS	GDM	T2D; GDM	MTNR1B (rs1387153 and rs10830963) is related to GDM
2	2020	Chinese	1,668	17	wGRS	Children’s overweight and obesity status; obesity-related quantitative traits	blood glucose	Among children of mothers without GDM, per SD increase in maternal GRS for blood glucose was positively associated with a 63% higher risk of childhood overweight and an 86% higher risk of childhood obesity
3	2020	American and Danes	2,434	59	SC-GRS	Postpartum diabetes	T2D	A higher GRS was significantly associated with a higher risk of T2D
4	2020	15 centers in nine countries	>5,000	390	wGRS	GDM; high weight of newborn	T2D; physiological characteristics of pregnancy	Positive correlation
5	2020	South Asian and participants living in the United Kingdom	832	14,971,357	P+T, LDpred, GraBLD	GDM	Glucose-related traits and T2D	The odds ratio of developing GDM was 2- to 2.5-fold higher in participants with the highest PRSs (top 25%) compared with the rest (75%) of the study population
6	2019	Asian	1,429	23	SC-GRS, DL-GRS	GDM	T2D	The risk of GDM correspondingly increases as the GRS increases (OR = 1.95, 95% CI: 1.46–2.61; OR = 1.99, 95% CI: 1.48–2.70)
7	2019	Pregnant women of European ancestry	1,939	31	SC-GRS	BMI during the gestational period	BMI	A GRS composed of BMI-associated genetic variants was associated with prepregnancy
8	2018	Indian	4,018	3	wGRS	Insulin secretion	Insulin secretion	GRS calculated based on the three SNPs associated with insulin resistance showed an increase in insulin resistance by 0.07 (SE = 0.145, *p* = 0.006) per allele
9	2018	15 centers in nine countries	1,931	150	wGRS	FG	FG	Positive correlation
10	2018	US population, the DNBC	8,722	11	SC-GRS, wGRS	GDM	T2D	The GRS based on the 11 SNPs was significantly associated with the risk of GDM
11	2018	Asian	1,156	10	wGRS	Weight changes during and after pregnancy (postpartum weight reduction and gestational weight gain)	HbA1c	A genetic risk score of HbA1c is related to long-term changes of HbA1c among women with preceding history of GDM
12	2018	Scandinavian ancestry	529	Publicly available GWAS data	wGRS	GDM	FG, 2hG, BMI, T2D	The GRS for FG (GRSFG) explained a similar amount of variance in FG in pregnancy at both measurement occasions as in the MAGIC consortium data
13	2017	Caucasian	1,996	34	SC-GRS, wGRS	GDM	T2D, GDM, glycemic traits	GRS, which included SNPs known to be associated with T2DM, was associated with the risk of GDM in Caucasians. The OR was 1.10 in SC-GRS (95% CI: 1.07–1.13) and 1.11 in wGRS (95% CI: 1.08–1.14)
14	2014	North America	296	36	SC-GRS, wGRS, explained variance GRS	GDM, prediabetes, and T2D among women with prior GDM	T2D	An explained variance GRS is associated with both GDM and progression to prediabetes and T2D in women with prior GDM
15	2014	55% Caucasian, 20% African-American, 16% Hispanic, 5% American Indian, and 4% Asian-American	3,234	34	SC-GRS	T2D; function of β cells	T2D	The GRS was positively associated with GDM history (OR = 1.05, 95% CI 1.00–1.08)
16	2013	South Korean	1,637	48	SC-GRS, wGRS	Postpartum diabetes	T2D	The GRS was higher in women who progressed to diabetes after a GDM pregnancy compared with those who showed NGT/IGT
17	2012	75% Europeans and 25% non-Europeans	793	13	wGRS	Postpartum diabetes	T2D	GRS including all these 13 SNPs was significantly associated with a higher risk of postpartum diabetes

T2D, type 2 diabetes mellitus; GDM, gestational diabetes mellitus; GRS, genetic risk score; PRS, polygenetic risk score; SNPs, single-nucleotide polymorphisms; GWAS, genome-wide association studies; SC-GRS, simple count genetic risk score; wGRS, weighted genetic risk score; DL-GRS, direct logistic regression genetic risk score; FG, fasting glucose; IGT, impaired glucose tolerance; NGT, normal glucose tolerance; 2hG, 2-h glucose; BMI, body mass index; SD, standard deviation; OR, odds ratio; CI, confidence interval; SE, standard error.

Kwak et al. found that the uGRS and wGRS were robustly associated with clinical parameters such as the effect of insulin treatment, fasting glucose, glucose AUC, and decreased insulin AUC. Moreover, they were significantly associated with a decreased insulin secretion/insulin resistance disposition index, which indicated impaired insulin secretory capacity (*β* = −0.025; *p* = 7.5 × 10^−5^). The wGRS can increase predictive power combined with other risk factors. Although the GRS has a limited value in identifying GDM at the population level in general, it can more precisely stratify women’s risk of GDM (23). The GRS could show genetic information of women at high risk for GDM to improve interventions so as to reduce the impact of metabolic disorders in pregnancy (24). A study identified eight variants associated with GDM based on the GRS (25). Nevertheless, a study showed that the GRS expresses a limited value in the identification of GDM cases (26).

### Relationship between GRS and the risk of T2DM in women with a history of GDM

A meta-analysis reported that women with a prior history of GDM had a more than sevenfold increased risk of developing T2DM 10 years after delivery compared with those with normal blood glucose (27). A study with long-term follow-up of two independent populations of White women with a history of GDM showed that the higher the GRS, the higher the T2D risk. Furthermore, the association may be related to dietary quality; the association between GRSs and the risk of T2D was stronger in pregnant women with a poor diet (28). Kwak et al. found that among women with a history of GDM, the GRSs of women who progressed to diabetes 1 year after delivery were significantly higher than those of women who progressed to normal or impaired glucose tolerance. Incorporating the wGRS into the model made up of risk factors such as age can improve the predictive power of the model (24). A GRS consisting of three previously reported SNPs associated with insulin secretion was not associated with the risk of GDM. Notably, the diagnostic criteria of insulin resistance are the WHO 2013 criteria (29). In women diagnosed with prediabetes, a GRS containing 34 diabetes-related loci was used to assess the genetic risk of diabetes, and it was found that women with a history of GDM had a significantly higher GRS than women without GDM (30).

### Relationship between GRS and adverse pregnancy outcomes

In addition, the GRS is associated with adverse pregnancy outcomes. Song et al. found that in the offspring of mothers with normal blood glucose during pregnancy, the risk of overweight and obesity increased by 63% and 86%, respectively, for each SD increase in the GRS. However, in children of GDM mothers, the same GRS model did not predict the risk of offspring being overweight or obese. The authors also found similarly significant interactions between genetically determined maternal blood glucose levels and GDM status on other obesity-related outcomes in children, such as body fat percentage (31). Han et al. found that compared with women with higher GRSs, women with lower GRSs showed more weight loss and better HbA1c improvement after delivery (32).

## Most common SNPs used in the GRS studies on GDM

To perform a comprehensive review, we searched PubMed using the terms “SNP” OR “Single nucleotide polymorphism” AND “gestational diabetes mellitus” OR “GDM,” “Genetic risk score” OR “GRS” OR “polygenetic risk score” OR “PRS” OR “risk score” AND “gestational diabetes mellitus” OR “GD” and included articles from January 2012 to May 2021. After excluding unrelated articles, only 17 reviews were included. We retrieved these articles that investigated the correlation between the GRS and GDM, from which we extracted all SNPs used to build the GRS model for each study. We sorted them according to the occurrence or frequency of each gene and found that the top 4 “high-frequency genes” most commonly used to build the GRS were *SLC30A8, TCF7L2*, *MTNR1B*, and *KCNJ11*.

### Transcription factor 7-like 2

Transcription factor 7-like 2 (*TCF7L2*) is a key transcriptional effector of the Wnt/beta-catenin signaling pathway, an important developmental signaling pathway that negatively regulates fat formation. It regulates insulin secretion and islet beta-cell proliferation/apoptosis and is a key gene in the regulation of glucose homeostasis (33).

Women carrying the *TCF7L2* rs7903146 risk alleles exhibit impaired insulin secretion and proinsulin conversion compared with non-carrier women (34). It is reported that even after adjusting for confounding factors such as BMI and age, the T risk allele in *TCF7L2* rs7903146 is associated with early postprandial glucose control failure and insulin treatment needs in women with gestational diabetes (35). The genetic variant rs7903146 (C > T) in *TCF7L2* presents a strong association with GDM risk (36). Another article presented evidence that *TCF7L2* rs290487, rs6585194, and rs7094463 polymorphisms were associated with insulin resistance and insulin secretion in women with GDM (37). Franzago et al. demonstrated an important correlation between the *TCF7L2* rs7903146 variant and GDM with a more than fivefold risk in the *TT* genotype (38).

On the contrary, another study found no statistically significant difference in glucose, cholesterol levels, and different *TCF7L2* SNP alleles in the GDM group. It is reported that *TCF7L2* SNPs are not different among women with GDM, although significantly higher incidences of *TCF7L2* rs7901695 SNP *CC/CT*, rs7903146 SNP *CT/TT*, and rs12255372 *GT/TT* were observed compared with the general female population (39). Similarly, Anghebem-Oliveira et al. found that *TCF7L2* rs7901695 was not associated with GDM in a Brazilian population (40).

### Melatonin receptor 1B gene

*MTNR1B*, the melatonin receptor 1B gene, encodes one of the receptors for melatonin and not only regulates circadian rhythm but also plays an important role in glucose metabolism, which is considered a bridge between circadian rhythm regulation and glucose metabolism (41).

Research has identified some SNPs utilizing GRS, including the risk SNPs rs10830963 (22, 25, 29, 32), rs1387153 (19, 22, 29), 2166706 (19), and rs7936247 (22) and two protective SNPs (rs1447352 and rs4753426) (19). Of these SNPs, rs10830963 is the most studied. A study indicated that *MTNR1B* rs10830963 is associated with GDM susceptibility and that women carrying the *G* allele have an increased risk of developing GDM (42). Among Chinese women with a history of GDM, Nisa et al. found that there was a bidirectional effect between *MTNR1B* gene variation and gestational weight gain in 2-h OGTT changes 1–5 years after delivery (43). It is suggested that the risk of GDM correlated with the rs10830963 risk allele *G* cannot be changed by other successful lifestyle interventions (44). Another article revealed that rs10830963, rs1387153, and rs2166706 interact with GDM risk in a southern Chinese population. rs1447352 and rs4753426, in particular, are associated with the reduction of the risk of GDM (45).

Another study revealed a strong association between rs1387153 and rs10830963 with GDM susceptibility in the dominant genotype (*p* = 0.006 and 0.007, respectively) and allelic (*p* = 0.008 and 0.013, respectively) models. Rosta et al. found that the *MTNR1B* rs10830963/*G* allele had the most robust association with GDM, as well as with glycemic traits, including both the FPG and the post-challenge (2 h) PG values at 75 g OGTT (46). Li et al. found that *MTNR1B* rs10830963 and its protein expression levels in the placenta are associated with an increased risk of developing GDM. Then, it may be a genetic factor leading to insulin resistance in Han Chinese women with GDM (47). The findings of another study suggested that rs10830963 and rs7936247 may be markers for susceptibility to GDM in a Chinese population (48). Firneisz et al. found that the rs10830963*/G* risk allele in *MTNR1B* was associated with a notable increase in the OR for antenatal insulin therapy initiation (OR = 5.2) in Hungarian women with GDM and pre-pregnancy BMI ≥29 kg/m^2^ (49).

### Zinc transporter-8 gene

The zinc transporter-8 gene (*SLC30A8*), which encodes insulin gland-specific zinc transporter-8, is an important factor in the regulation of insulin secretion in islet cells that can promote zinc accumulation in the vesicles of islet β cells*. A* study showed that in women with gestational weight gain of GDM, *SLC30A8* rs13266634 has a certain influence on long-term blood glucose changes, and women carrying the rs13266634 *C* allele will be limited in weight gain during pregnancy, which will reduce the occurrence of postpartum hyperglycemia (50). In the population of the district of Lund, the rs13266634 *C* allele in *SLC30A8* was associated with an increased risk of developing GDM (51). Another study suggested that *SLC30A8* rs13266634/*T* is a protective variant against the development of GDM among European women (46). *SLC30A8* rs13266634/*T* also showed significant associations with GDM among Filipinos (52). Furthermore, *SLC30A8* rs3802177 was also significantly associated with the risk of GDM (25). In Han Chinese, the *CC* genotype of *SLC30A8* rs2466293 was significantly correlated with an increased risk of GDM with an OR of 1.455 (95% CI: 1.077, 1.966; *p*-value = 0.014) after adjustment for age. It was also significantly associated with FPG and 2hPG during OGTT (53).

### Potassium channel inwardly rectifying subfamily J member 1

Potassium channel inwardly rectifying subfamily J member 11, also called *KCNJ11*, located on chromosome 11p15.1, is an open-reading frame encoding an inward rectifying potassium channel Kir6.2 protein composed of 390 amino acids. It is important for insulin secretion (54). A study revealed that *KCNJ11* rs5219 was associated with GDM (OR = 1.15; 95% CI: 1.06, 1.24; *p* = 0.0004) (41). Another meta-analysis presented that the *T* allele of rs5219 was associated with an increased risk of GDM (pooled OR = 1.15; 95% CI: 1.06, 1.26) (55). A study showed that in Chinese women, gestational glycemic traits, such as HOMA-IR, insulin disposition index, early-phase insulin release, and 2-h postprandial proinsulin conversion, were associated with the risk allele of *KCNJ11* rs5219 (*p* = 0.001, 0.006, 0.001, and 4.2 × 10^−12^, respectively) (56). *KCNJ11* rs5215 is related to the development of postpartum abnormal glucose tolerance in Japanese women (OR = 1.82, 95% CI: 1.05, 3.14; *p* = 0.032) (57). However, no evidence was found on the association between *KCNJ11* rs5219 and susceptibility to GDM or to other relevant metabolic features in a Greek population (58). Similarly, a non-significant association was found between *KCNJ11* and the risk of GDM in a Chinese population (59).

## Conclusion and future prospects

As GDM has become a common but difficult complication of pregnancy, it has received increasing research attention. Its harm to the puerpera and newborns cannot be ignored. Early detection and prevention are key to managing GDM. The GRS provides a way to solve this problem. While a large number of studies have shown that for such complex diseases, GDM-related risk genes can be identified through genetic methods such as GWAS and GRSs, the predictive value of relevant prediction models is limited; more clinical factors and indicators may need to be combined to obtain sufficient results. Some pregnant women develop GDM even without traditional risk factors such as family history. Therefore, the combinations of clinical characteristics that can build a reliable prediction model of GDM-related diseases are an urgent research problem to be explored and solved. The studies described in this article used GRSs to predict the development of T2D and GDM, determine their associations with disease diagnosis and prognosis, provide new evidence for risk assessment and early diagnosis of GDM, and identify their adverse consequences. However, it is shown that the development of GRSs is associated with some risks, such as incorrect risk estimation for individuals, failure to convey uncertainty in the assessment, and aggravating genetic discrimination (60). The cost-effectiveness of carrying out GRSs is also not negligible. Currently, most GWAS are conducted using Caucasian individuals, the utilization of these SNPs in other populations may result in a poor predictive power of GRSs, and then LD differences may be a major challenge to overcome (61).

Utilizing GRSs appropriately is full of challenges and opportunities. Improving the diagnosis and treatment of GDM solely based on the genetic background is insufficient; thus, the accuracy of predictions can be improved by combining other known risk factors, such as age and diet, among others. The gaps in GRS research need to be filled in order to use GRSs more effectively and safely before they can be used on a large scale. The epidemiology of large population-based cohort studies and the evaluation of the effectiveness of predictions in large populations will advance personalized medicine and ultimately improve health among susceptible populations.

## Author contributions

YT wrote the first draft of the manuscript and edited it. PL performed critical revision of the literature and edited the manuscript. All authors contributed to the article and approved the submitted version.

## Funding

This article was funded by the Natural Science Foundation of Liaoning Province (2020-MS-153).

## Conflict of interest

The authors declare that the research was conducted in the absence of any commercial or financial relationships that could be construed as a potential conflict of interest.

## Publisher’s note

All claims expressed in this article are solely those of the authors and do not necessarily represent those of their affiliated organizations, or those of the publisher, the editors and the reviewers. Any product that may be evaluated in this article, or claim that may be made by its manufacturer, is not guaranteed or endorsed by the publisher.

## Abbreviations

GRS, genetic risk score; PRS, polygenetic risk score; SNPs, single-nucleotide polymorphisms; GWAS, genome-wide association studies; SC-GRS, simple count genetic risk score; OR, odds ratio; P + T, pruning and thresholding; DL-GRS, direct logistic regression GRS; 2hG, 2-h glucose; LD, linkage disequilibrium; FG, fasting glucose; IGT, impaired glucose tolerance; NGT, normal glucose tolerance; BMI, body mass index; SD, standard deviation; CI, confidence interval; UGRS, unweighted GRS; GraBLD GRS, gradient boosted and linkage disequilibrium adjusted GRS; SE, standard error.
